# Giant hypervascular atypical lipomatous tumor/well-differentiated liposarcoma of the cervicodorsal region: a case report

**DOI:** 10.3389/fmed.2026.1925841

**Published:** 2026-10-21

**Authors:** Zhonghai Liao, Feng Qin, Song Wang, Xinyu Wan, Hong Li

**Affiliations:** 1 Department of Burns and Plastic Surgery, North Sichuan Medical College, School of Clinical Medicine, Nanchong, Sichuan, China; 2 Department of Burns and Plastic Surgery, West China School of Medicine, Sichuan University, Sichuan University Affiliated Chengdu Second People’s Hospital, Chengdu Second People’s Hospital, Chengdu, Sichuan, China; 3 Department of Medical Cosmetology, West China School of Medicine, Sichuan University, Sichuan University Affiliated Chengdu Second People’s Hospital, Chengdu Second People’s Hospital, Chengdu, Sichuan, China

**Keywords:** atypical lipomatous tumor, CDK4, cervicodorsal mass, computed tomography angiography, MDM2, soft tissue sarcoma, well-differentiated liposarcoma

## Abstract

We report a case of giant hypervascular atypical lipomatous tumor/well-differentiated liposarcoma (ALT/WDLPS) of the cervicodorsal region in a 62-year-old man. The mass had enlarged slowly over 17 years, with accelerated growth during the preceding 2 years accompanied by a local skin pulling sensation, but without definite airway, neurologic, or major vascular compression. Preoperative computed tomography angiography (CTA) demonstrated a lesion of approximately 20.9 cm × 28.6 cm × 25.4 cm containing internal septa, nodular components, and marked vascularity supplied predominantly by branches of both subclavian arteries. The patient underwent *en bloc* tumor excision with local advancement flap reconstruction; the resected specimen weighed approximately 6.01 kg, the resulting defect measured approximately 15 cm × 11 cm, the operative time was nearly 6 h, and the estimated blood loss was approximately 700 mL. Postoperative pathology showed focal spindle-cell proliferation with myxoid change and, together with the MDM2, CDK4, and p16 positivity documented in the pathology report, supported the diagnosis of ALT/WDLPS. At short-term follow-up, the incision and flaps had healed well, with no definite local recurrence. This case indicates that a long-standing, slowly enlarging giant lipomatous mass in the cervicodorsal region should not be excluded from the diagnosis of ALT/WDLPS solely on the basis of indolent clinical behavior. When a lesion is very large or imaging suggests rich vascularity, preoperative vascular assessment, hemostatic contingency planning, and simultaneous wound reconstruction design should be incorporated into the surgical plan.

## Introduction

The differential diagnosis of a giant mass in the shoulder, neck, and upper back is broad, encompassing lipoma, neurogenic tumor, hemangioma, lymphangioma, and other soft tissue neoplasms. Although most lipomatous masses are benign lipomas, atypical lipomatous tumor/well-differentiated liposarcoma (ALT/WDLPS) should be suspected when a lesion demonstrates long-standing growth, very large size, poorly defined margins, or involvement of an anatomically complex region such as the posterior neck and upper back. Liposarcomas account for approximately 5%–6% of all soft tissue sarcomas and most commonly arise in the retroperitoneum and deep soft tissues of the extremities; involvement of the head and neck is comparatively rare (1, 2). ALT/WDLPS is a low-grade adipocytic neoplasm whose imaging characteristics may overlap with those of lipoma. Computed tomography (CT) and magnetic resonance imaging (MRI) are valuable for assessing tumor extent, tissue composition, relationship to adjacent structures, and surgical resectability (3, 4). Definitive diagnosis typically depends on histopathologic examination supplemented by immunohistochemistry or molecular testing, including evaluation for MDM2 and CDK4 expression (5).

Surgical excision remains the cornerstone of treatment for ALT/WDLPS in the head and neck, and requires a balance between oncologic safety, protection of important neurovascular structures, and wound reconstruction. Previous reports of posterior neck and laterocervical ALT/WDLPS have emphasized the typically slow growth, large size, and frequent preoperative misdiagnosis as lipoma (6, 7), whereas descriptions of preoperative vascular assessment, intraoperative hemorrhage control, and simultaneous reconstruction of extensive defects have been relatively limited. We herein present a giant hypervascular ALT/WDLPS of the cervicodorsal region, with particular focus on vascular assessment, surgical excision, simultaneous reconstruction, and pathologic differentiation.

## Case presentation

### Clinical presentation and preoperative assessment

A 62-year-old man was admitted with a chief complaint of a back mass present for more than 17 years that had enlarged markedly over the preceding 2 years. The patient initially noticed a subcutaneous mass on his back without identifiable precipitating factors. At onset, the lesion was approximately the size of a quail egg and was accompanied by occasional pruritus, without pain, numbness, ulceration, or hemorrhage. The mass enlarged gradually over the ensuing years. During the 2 years preceding admission, growth accelerated substantially, progressively interfering with daily activities, and the patient described a marked pulling sensation involving the occipital scalp and cervicodorsal skin. He denied fevers, night sweats, weight loss, dyspnea, dysphagia, hoarseness, upper extremity numbness or weakness, or other neurologic deficits. His past medical history was unremarkable for trauma, prior surgery, or significant systemic disease.

On admission, vital signs were stable. Physical examination revealed a giant, diffuse mass extending from the posterior neck to the upper back, producing a prominent hump-like contour with a surface span exceeding 20 cm. The overlying skin was largely normal in color but markedly distended, with scattered cutaneous pigmentation, multiple small papules, and a few superficial varicosities, but no erythema, ulceration, or scarring. The mass had poorly defined margins, soft consistency, and no significant tenderness; it was not adherent to the overlying skin and demonstrated limited mobility. No clinical signs of airway compression, major cervical vascular compression, or neurologic involvement were identified (Figure 1).

**Figure 1:**
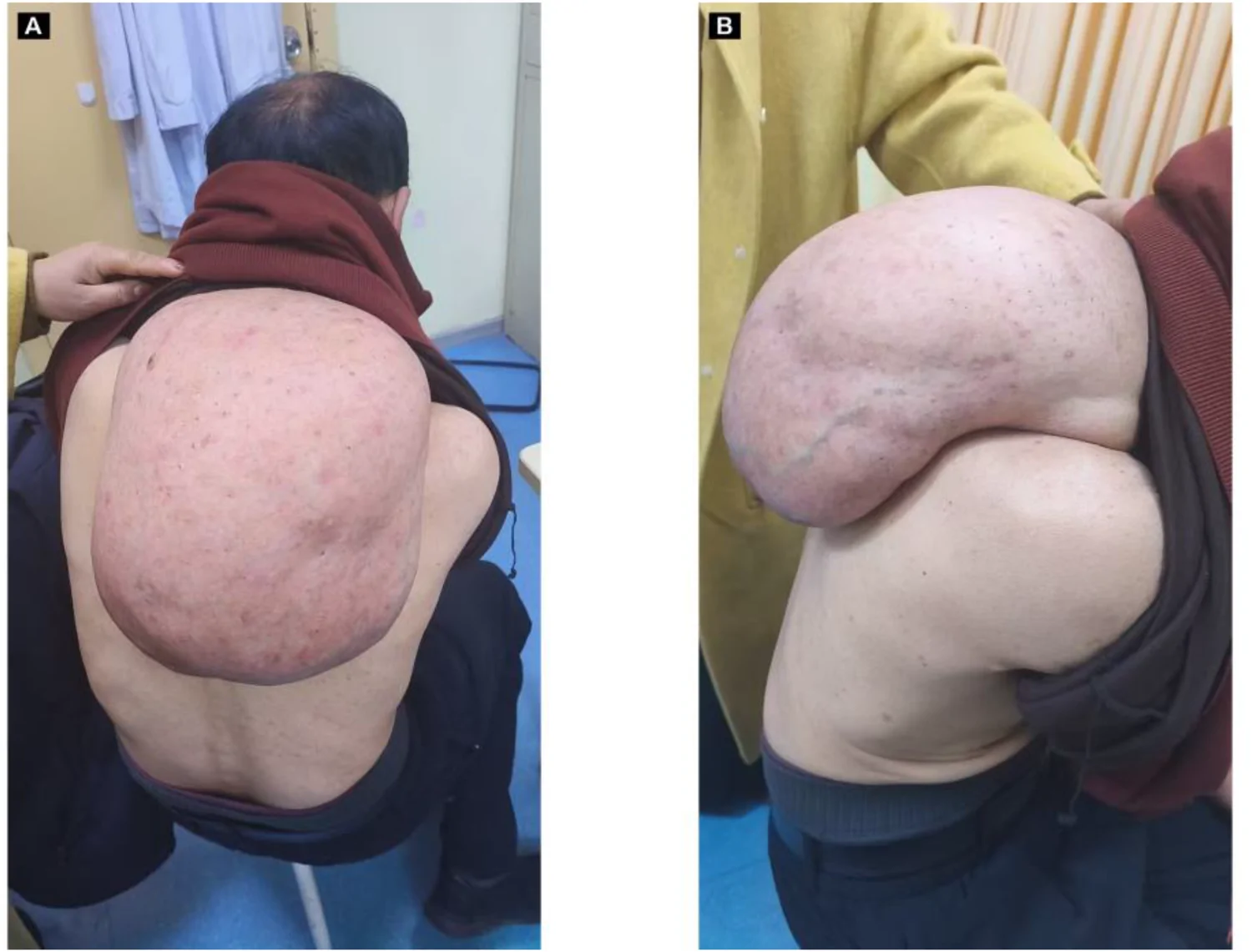
Preoperative appearance of the cervicodorsal mass. **(A)** Posterior view showing the giant hump-like prominence extending from the posterior neck to the upper back. **(B)** Posterolateral view showing the tumor protruding markedly from the body surface, with stretched overlying skin and superficial veins.

Preoperative CTA demonstrated a giant subcutaneous fat-density lesion in the nuchal and upper dorsal region, measuring approximately 20.9 cm × 28.6 cm × 25.4 cm, with indistinct margins. Multiple internal septa, small patchy hazy areas, nodular soft-tissue components, and focal uneven cutaneous thickening were identified. Vascular assessment demonstrated marked hypervascularity, with the principal arterial supply derived from branches of both subclavian arteries, including the right deep cervical artery, the right superficial cervical artery, and branches of the left superficial cervical artery, accompanied by numerous tortuous small vessels (Figure 2).

**Figure 2:**
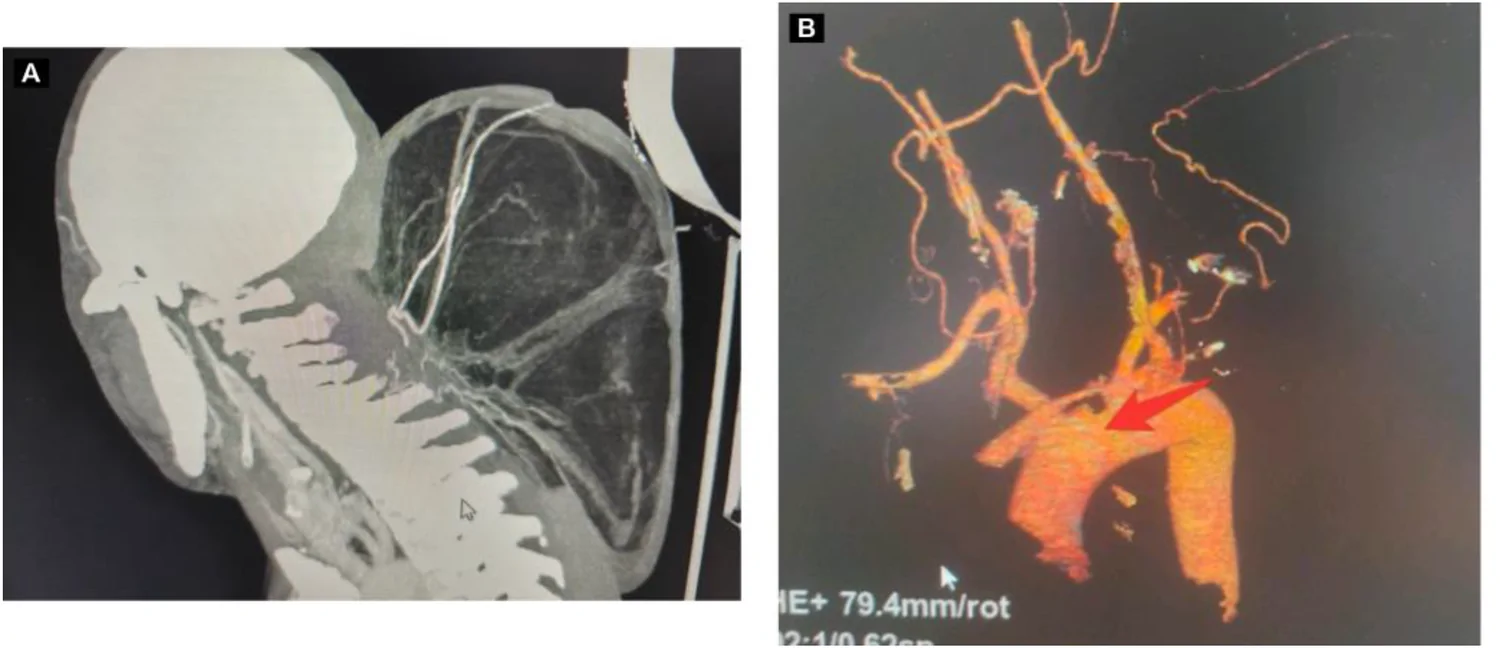
Preoperative computed tomography angiography (CTA). **(A)** Sagittal reconstruction showing a giant subcutaneous lipomatous cervicodorsal mass with internal septa and enhancing vessels. **(B)** Three-dimensional volume-rendered reconstruction showing tortuous tumor-feeding vessels arising mainly from branches of both subclavian arteries; red arrows indicate the regions of the feeding vessels.

Preoperative local biopsy submitted two pieces of skin tissue that showed no specific abnormality, together with one additional specimen composed predominantly of mature adipose tissue, requiring initial differentiation between ordinary lipoma and ALT/WDLPS. Limited local sampling may not represent the internal heterogeneity of such a giant tumor; in view of the accelerated growth over the preceding 2 years, the poorly defined margins, and the septa and nodular components demonstrated on imaging, ALT/WDLPS remained a preoperative concern, and further evaluation of the completely resected specimen was required.

### Surgical treatment and wound reconstruction

After comprehensive preoperative evaluation, the patient underwent excision of the giant cervicodorsal mass and reconstruction with local advancement flaps under general anesthesia with endotracheal intubation in the prone position. Operative space in the cervicodorsal region was limited, and the massive tumor obscured the surgical field, increasing the difficulty of vascular exposure and hemostasis. Incisions and advancement flaps were designed preoperatively according to the tumor extent, the skin to be preserved, and the anticipated defect, with care to protect the blood supply of the planned flaps. Dissection proceeded along the interface between the tumor periphery and deep surface and the underlying tissues. No gross muscle invasion was observed intraoperatively, and there was no definite adhesion between the tumor and major neurovascular structures. Multiple large feeding vessels and a broad oozing surface were encountered at the tumor base; guided by the vascular distribution shown on CTA, the bilateral main feeding arteries were exposed when approximately two-thirds of the tumor had been dissected and were ligated individually, and bleeding at the base was controlled with electrocautery and suture ligation. Because continued inferior-to-superior dissection increased pressure on the neck and face owing to the tumor's volume and weight, the dissection direction was changed to superior-to-inferior while capsular continuity was maintained as far as possible, and the tumor was ultimately removed *en bloc* (Figures 3,4).

**Figure 3:**
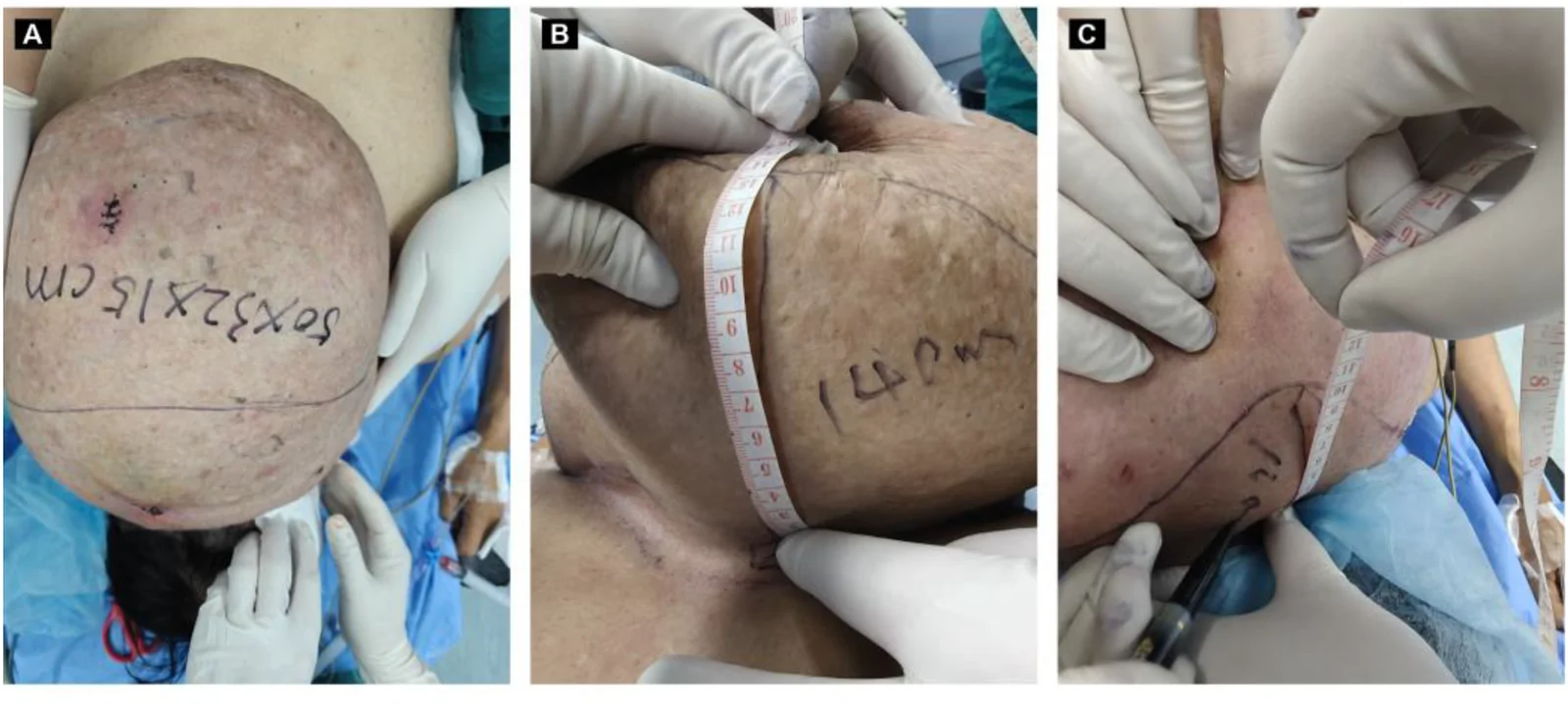
Preoperative surface measurement and skin preservation assessment. **(A)** Marking of the tumor extent, incision, and skin to be preserved in the prone position. **(B,C)** Measurement from different directions of the preservable skin and its advancement range, providing the basis for local advancement flap design after excision.

**Figure 4:**
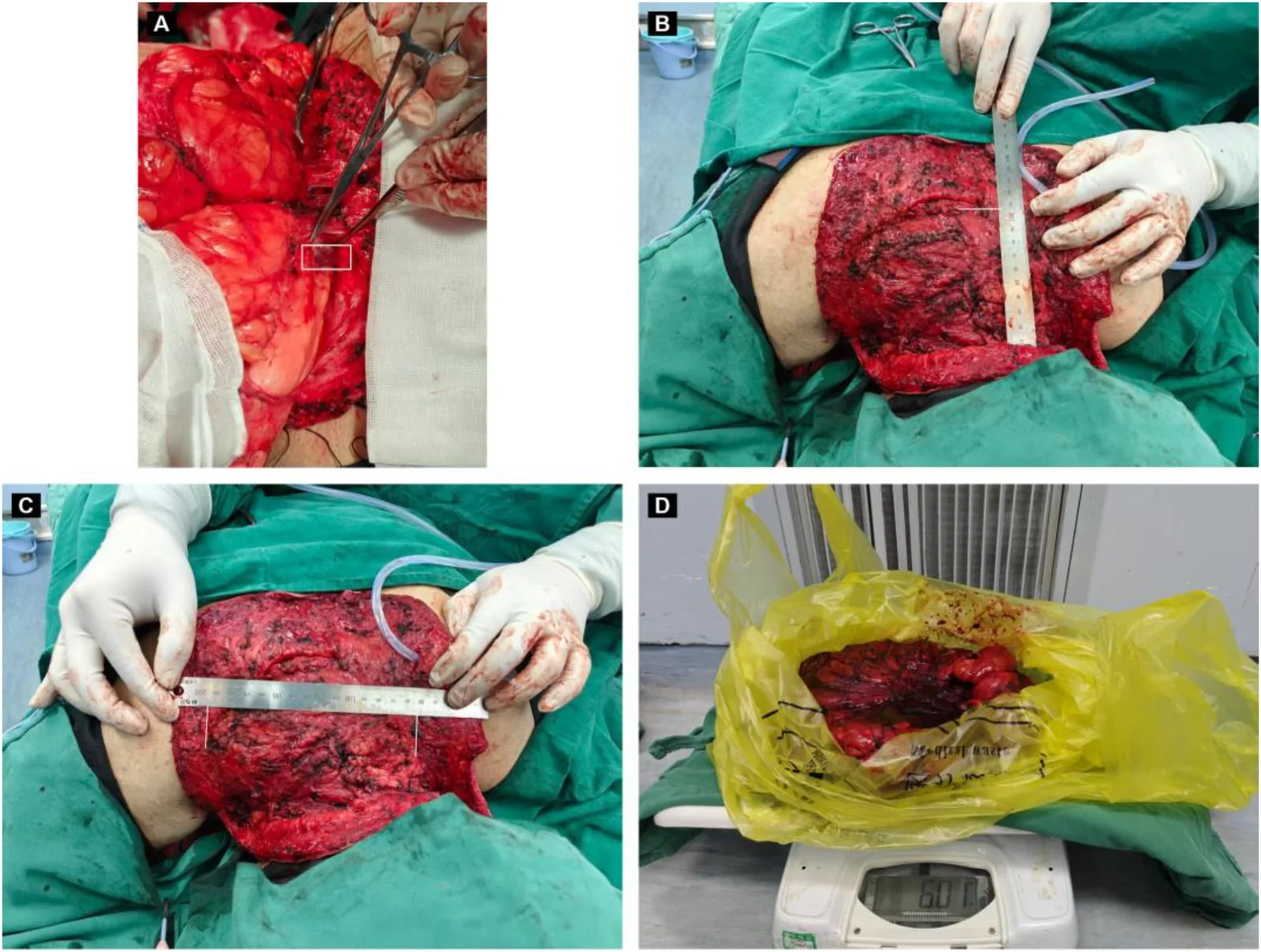
Intraoperative vessels, post-excision defect, and resected specimen. **(A)** Main feeding vessels at the tumor base; the white box indicates a representative vessel. **(B,C)** Measurement of the longitudinal and transverse dimensions of the post-excision defect, approximately 15 cm × 11 cm. **(D)** The *en bloc* resected specimen placed on an electronic scale, with a net weight of approximately 6.01 kg.

After tumor removal, an irregular defect of approximately 15 cm × 11 cm remained. Following meticulous hemostasis, the reserved local skin and soft tissue were advanced to cover the defect, and redundant wound edges were trimmed as needed according to wound edge apposition before closure. One drainage tube was placed in the residual cavity beneath the flap and brought out through the patient's left wound edge. The operative time was nearly 6 h, the estimated intraoperative blood loss was approximately 700 mL, and no transfusion was required. Postoperative drainage and adjunctive local negative-pressure wound therapy were applied to reduce residual dead space and fluid accumulation and to promote flap apposition. The patient recovered uneventfully and was discharged 12 days after surgery (Figures 4, 5).

**Figure 5:**
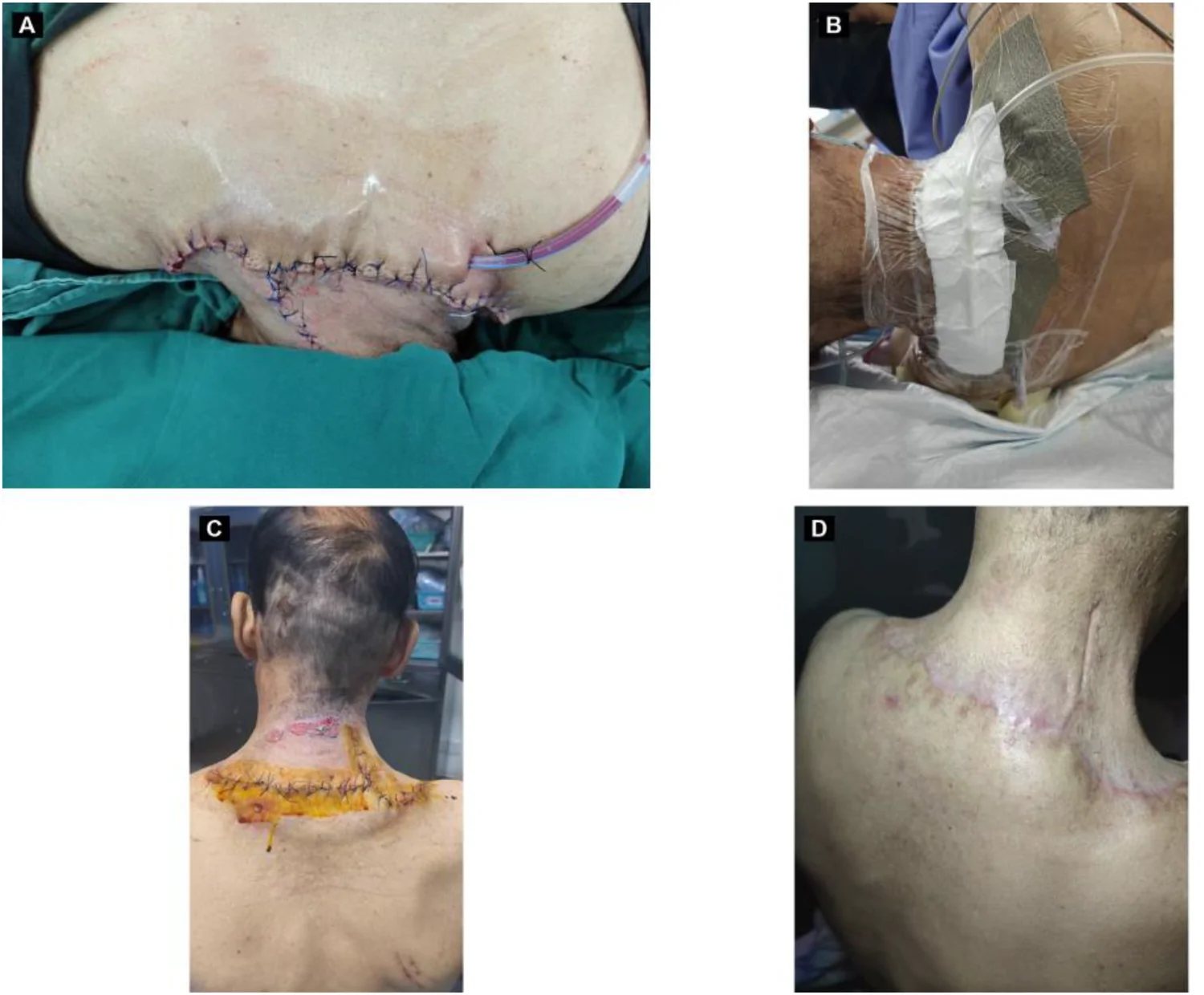
Local advancement flap reconstruction and postoperative follow-up. **(A)** Immediate appearance after completion of flap advancement reconstruction, with the drainage tube exiting through the patient's left wound edge. **(B)** Postoperative adjunctive local negative-pressure wound therapy. **(C)** Appearance of the incision and flap 10 days postoperatively. **(D)** Incision healing and scar appearance 6 months postoperatively.

### Pathologic findings and follow-up

Preoperative local biopsy submitted three tissue specimens in total: two skin specimens showed no specific abnormality, and one specimen consisted predominantly of mature adipose tissue, indicating a lipomatous neoplasm and requiring initial differentiation between ordinary lipoma and atypical lipomatous tumor/well-differentiated liposarcoma (ALT/WDLPS). Given the enormous tumor volume and the septa and nodular non-fatty components demonstrated on imaging, the preoperative biopsy findings together with the imaging features indicated that further evaluation of the completely resected specimen was necessary.

Postoperative pathologic examination showed that the completely resected specimen was a giant lipomatous tumor measuring approximately 37 cm × 25 cm × 8.5 cm. Histologically, the tumor was composed predominantly of mature adipocytes of varying sizes separated by widened fibrous septa; scattered atypical stromal cells with enlarged, hyperchromatic nuclei were present within the septa, together with focal spindle-cell proliferation and myxoid stromal change. Immunohistochemical studies demonstrated positivity for S-100, p16, MDM2, and CDK4; CD34 was negative; RB1 expression was retained; and the Ki-67 proliferative index was approximately 5%. Integrating the preoperative biopsy, the histologic features of the complete resection specimen, and the immunophenotype, the pathologic diagnosis supported ALT/WDLPS. MDM2 fluorescence *in situ* hybridization (FISH) was recommended but not performed. Margin assessment showed that tumor tissue approached the inked deep margin, with a closest distance of approximately 0.5 mm; no tumor cells touched the ink, and the deep margin was therefore classified as negative but close (Figure 6).

**Figure 6:**
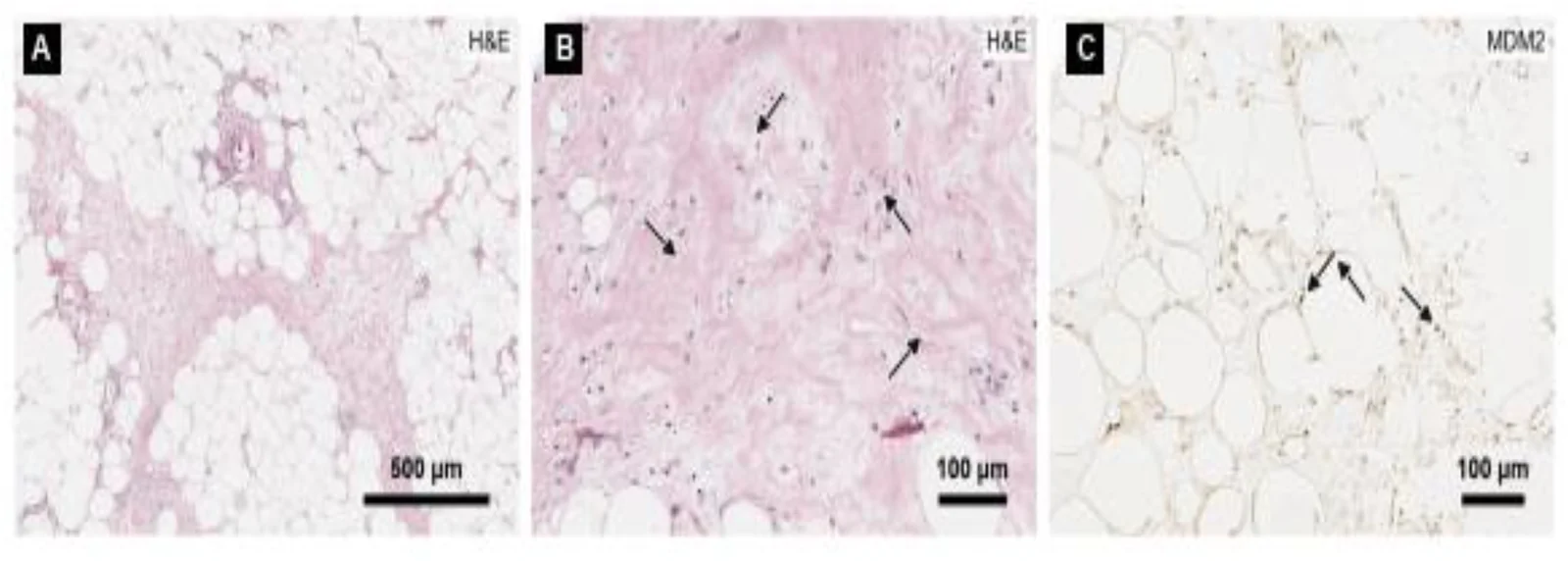
Histologic and immunohistochemical features of ALT/WDLPS. **(A)** Hematoxylin and eosin (H&E) low-to-intermediate-power view showing mature adipocytes and widened, thickened fibrous septa. **(B)** H&E high-power view showing scattered atypical stromal cells with enlarged, hyperchromatic nuclei within the fibrous septa (arrows). **(C)** MDM2 immunohistochemical staining showing nuclear staining of scattered stromal cells (arrows). Scale bars: a, 500 μm; b, c, 100 μm.

At 6 months postoperatively, the patient remained in good general condition. The cervicodorsal incision and flaps had healed well, with no flap necrosis, wound dehiscence, infection, hematoma, or significant fluid collection. The cervicodorsal contour and performance of daily activities were improved compared with before surgery, and shoulder and neck range of motion was not significantly limited. Follow-up color Doppler ultrasonography revealed no definite abnormal echogenicity or abnormal blood flow signals at the bilateral cervical scar regions. Taken together with the physical examination findings, no definite evidence of local recurrence was identified during the follow-up period. In view of the negative but close deep margin, continued regular follow-up was recommended, with particular attention to local recurrence, wound and flap status, and shoulder-neck functional assessment (Figure 5).

## Discussion

Previous cases of posterior neck and laterocervical ALT/WDLPS indicate that slow growth, the absence of significant symptoms, and fat-predominant imaging appearances may all lead to preoperative underestimation. The posterior neck ALT/WDLPS resection specimen reported by Almotairi et al. measured 14 cm × 13.5 cm × 5.5 cm, and preoperative CT and MRI had suggested an intermuscular lipoma; the laterocervical ALT/WDLPS reported by Komnos et al. measured 8 cm × 6 cm × 2.5 cm, and preoperative fine-needle aspiration yielded only adipose tissue (6, 7). As a benign reference, Du et al. reported a giant posterior neck lipoma with a disease course of 10 years and a resection specimen measuring 20 cm × 18 cm × 8 cm, which likewise required excision of redundant skin and drain placement with negative-pressure drainage (8). These cases illustrate that a long disease course, large size, and the need for skin trimming are not specific manifestations of ALT/WDLPS, and that adequate sampling and pathologic evaluation remain the key to distinguishing benign from malignant lesions. Compared with the above cases, the resection specimen in the present case was larger (37 cm × 25 cm × 8.5 cm; 6.01 kg), the disease course reached 17 years, and bilateral rich vascular supply coexisted with the need to reconstruct an approximately 15 cm × 11 cm defect; its complexity lay mainly in the combination of early diagnostic uncertainty, vascular risk, and the need for simultaneous reconstruction.

Histopathologic morphology and molecular testing are central to establishing the diagnosis of ALT/WDLPS. Kilpatrick's review noted that ALT/WDLPS shows morphologic overlap with ordinary lipoma, spindle-cell/pleomorphic lipoma, and atypical spindle-cell/pleomorphic lipomatous tumor, and that challenging cases require combined evaluation with MDM2, CDK4, and corresponding molecular testing (5). Using FISH results as the reference standard, Nomura et al. found that ALT/WDLPS diagnoses previously made on the basis of HE morphology alone tended toward overdiagnosis, and that the diagnostic accuracy of MDM2 and CDK4 immunohistochemistry was also limited (9). Gambella et al. further summarized the amplification, non-amplification, and polysomy patterns of MDM2 FISH and cautioned that inconsistent interpretation criteria may lead to misinterpretation (10). These studies indicate that morphologic, immunophenotypic, and molecular evidence should be interpreted in a tiered manner. In the present case, the morphologic features of the complete resection specimen and the positivity for MDM2, CDK4, and p16 support ALT/WDLPS; however, because MDM2 FISH was not completed, the current diagnosis is one made with the support of histomorphology and immunohistochemistry.

In the differential diagnosis, ordinary lipoma typically lacks atypical stromal cells with enlarged, hyperchromatic nuclei within fibrous septa and is generally not co-positive for MDM2 and CDK4; therefore, the histology and immunophenotype of this case do not support ordinary lipoma. Spindle-cell lipoma and atypical spindle-cell/pleomorphic lipomatous tumor more commonly show CD34 expression and RB1 loss, whereas the present case was CD34-negative with retained RB1 expression and concurrent MDM2 and CDK4 positivity, making these entities less likely (5). Myxoid liposarcoma is typically composed of primitive spindle cells within a myxoid matrix, delicate branching capillaries, and lipoblasts, and harbors DDIT3-related fusions; the present case showed only focal myxoid change without this typical morphologic pattern and therefore does not support myxoid liposarcoma (11). Integrating the preoperative biopsy, the histologic features of the complete resection specimen, and the supplementary immunohistochemical results, the pathologic diagnosis supports ALT/WDLPS.

Imaging can be used to identify suspicious features in lipomatous tumors, but its discriminatory ability has clear boundaries. Wilson et al., including 26 studies with a total of 2,613 patients, found a pooled sensitivity of 85% and specificity of 63% for overall radiologic assessment, indicating that CT and MRI can identify most suspicious lesions but still carry a certain false-positive rate (12). The systematic review by Muhib et al. focusing on ordinary lipoma versus ALT/WDLPS likewise noted that although larger size, thick septa, enhancement, and non-fatty components can raise the level of suspicion, the imaging appearances of the two entities still overlap (13). Therefore, imaging is better suited to detecting suspicious features, assessing lesion extent, and performing risk stratification, and cannot replace histologic diagnosis.

Multidimensional comprehensive assessment may improve preoperative identification. Asano et al. combined tumor site, size, depth, and septation and enhancement on MRI with histopathologic findings to establish a comprehensive scoring system for differentiating ordinary lipoma from ALT/WDLPS (14). This study illustrates that no single clinical feature, limited sampling result, or imaging sign should be interpreted in isolation from other evidence. The present case showed long-standing, slow, painless growth, and the local biopsy consisted predominantly of mature adipose tissue, resembling ordinary lipoma; however, the accelerated growth over the preceding 2 years, the enormous size, the poorly defined margins, and the multiple septa and nodular non-fatty components on imaging suggested that limited sampling might underestimate the lesion. These features constitute a rationale for obtaining the complete specimen for pathologic evaluation, but they cannot by themselves confirm ALT/WDLPS.

Current soft tissue tumor imaging guidelines recommend MRI as the first-line modality for tissue characterization and local staging of most lesions (15). Accordingly, CTA should not replace MRI for tissue characterization; rather, when a lesion is very large, appears hypervascular, or requires clarification of the dominant vascular supply before surgery, its more appropriate role is targeted vascular assessment, providing anatomic information for the surgical approach, vascular handling, hemostatic preparation, and reconstruction design. In the present case, CTA showed that the lesion was supplied by branches related to both subclavian arteries, including the right deep cervical artery, the right superficial cervical artery, and branches of the left superficial cervical artery; accordingly, exposure and ligation of the bilateral feeding vessels and protection of flap perfusion were incorporated into the preoperative plan, and multiple large feeding vessels were indeed encountered intraoperatively and ligated individually after exposure. The change from inferior-to-superior to superior-to-inferior dissection was an intraoperative adjustment made because the tumor's volume and weight increased pressure on the neck and face, and was not directly determined by CTA. Thus, this case demonstrates that CTA can provide surgically relevant vascular information, but it cannot prove that CTA reduces blood loss or improves long-term oncologic outcomes.

Previous studies provide limited clues regarding enhancement patterns and angiogenesis in lipomatous tumors. The MR imaging-pathology correlation study by Hosono et al. found that some enhancing thickened septa in liposarcoma may contain muscular components (16); Achilles et al. observed a correlation between tumor volume and microvessel density in an experimental human liposarcoma mouse model (17). The former cannot be used to infer muscle invasion in a specific case, and the latter is not an ALT/WDLPS clinical cohort; moreover, microvessel density and the feeding vessels visible on CTA are not the same measure. Therefore, these studies can only suggest possible histologic or biologic links and cannot be used to predict dedifferentiation, recurrence, or growth rate from CTA findings. In the present case, CTA showed rich vascularity, and no gross muscle invasion was observed intraoperatively; the available evidence supports using this finding only for vascular and hemostatic planning and does not support further tumor-biology inference.

Surgery remains the main treatment modality for resectable ALT/WDLPS. Recent UK guidelines for the management of soft tissue sarcomas and surgical cohorts of extremity and trunk ALT/WDLPS support complete resection balancing local control and functional preservation in appropriate patients, but the relevant local recurrence data derive mainly from non-head-and-neck cases and cannot be directly extrapolated (18, 19). Regarding adjuvant radiotherapy, a retrospective multicenter study of extremity ALT/WDLPS showed that it was associated with lower recurrence rates but more complications, and the median time to recurrence among patients who recurred was 8.2 years (20). This evidence supports individualized decision-making based on margin status, recurrence risk, the cost of re-excision, and treatment-related harm, and emphasizes long-term follow-up. The present case was managed with *en bloc* resection, ligation of the bilateral main feeding vessels, and local advancement flap reconstruction, with drainage and adjunctive local negative-pressure therapy to reduce residual dead space and fluid accumulation. The deep margin was negative but close, with a closest distance of approximately 0.5 mm; subsequent management should be assessed in light of pathologic review, local imaging, the functional cost of re-excision, and the patient's situation, and adjuvant radiotherapy should not be decided solely on the basis of a close margin.

Taken together, the significance of this case is not to prove the effectiveness of any single test or treatment strategy, but to add clinical decision-making experience under the conditions of a special anatomic site and enormous tumor burden. Previous cases have shown that posterior neck and laterocervical ALT/WDLPS can be underestimated because of lipoma-like appearances or limited sampling; imaging, pathology, and treatment studies have respectively defined the evidence boundaries of risk stratification, definitive diagnosis, and local control. Building on this foundation, the present case further demonstrates a continuous management process extending from limited biopsy, pathologic diagnosis of the complete specimen, and targeted vascular assessment to *en bloc* resection and simultaneous wound reconstruction, and particularly reflects the preoperative planning needs when bilateral vascular supply and a large defect coexist. Its value therefore lies mainly in providing a reference diagnostic and treatment approach rather than forming directly generalizable conclusions about efficacy.

## Conclusion

Even when a giant cervicodorsal lipomatous mass has grown slowly over a prolonged period, differentiation of ALT/WDLPS on the complete resection specimen remains necessary. For lesions with suspected rich vascularity, CTA can supplement information on the vascular supply and help plan vascular handling and hemostasis, and the need for wound reconstruction should be assessed synchronously before resection. In the present case, the morphology and immunophenotype of the complete specimen support ALT/WDLPS, but molecular confirmation of MDM2 gene amplification is still lacking. The surgical defect in this case was repaired with local advancement flaps, achieving favorable early outcomes.

## Data Availability

The original contributions presented in the study are included in the article/Supplementary Material, further inquiries can be directed to the corresponding author.
